# DNMT3L Modulates Significant and Distinct Flanking Sequence Preference for DNA Methylation by DNMT3A and DNMT3B *In Vivo*

**DOI:** 10.1371/journal.pgen.1001106

**Published:** 2010-09-09

**Authors:** Bethany L. Wienholz, Michael S. Kareta, Amir H. Moarefi, Catherine A. Gordon, Paul A. Ginno, Frédéric Chédin

**Affiliations:** Department of Molecular and Cellular Biology, University of California Davis, Davis, California, United States of America; The Babraham Institute, United Kingdom

## Abstract

The DNTM3A and DNMT3B *de novo* DNA methyltransferases (DNMTs) are responsible for setting genomic DNA methylation patterns, a key layer of epigenetic information. Here, using an *in vivo* episomal methylation assay and extensive bisulfite methylation sequencing, we show that human DNMT3A and DNMT3B possess significant and distinct flanking sequence preferences for target CpG sites. Selection for high or low efficiency sites is mediated by the base composition at the −2 and +2 positions flanking the CpG site for DNMT3A, and at the −1 and +1 positions for DNMT3B. This intrinsic preference reproducibly leads to the formation of specific *de novo* methylation patterns characterized by up to 34-fold variations in the efficiency of DNA methylation at individual sites. Furthermore, analysis of the distribution of signature methylation hotspot and coldspot motifs suggests that DNMT flanking sequence preference has contributed to shaping the composition of CpG islands in the human genome. Our results also show that the DNMT3L stimulatory factor modulates the formation of *de novo* methylation patterns in two ways. First, DNMT3L selectively focuses the DNA methylation machinery on properly chromatinized DNA templates. Second, DNMT3L attenuates the impact of the intrinsic DNMT flanking sequence preference by providing a much greater boost to the methylation of poorly methylated sites, thus promoting the formation of broader and more uniform methylation patterns. This study offers insights into the manner by which DNA methylation patterns are deposited and reveals a new level of interplay between members of the *de novo* DNMT family.

## Author Summary

The methylation of cytosine bases in DNA represents an extra layer of heritable biological information necessary for regulating gene expression and ensuring genomic stability in mammals. In this paper, we examine the function of the proteins responsible for laying down the initial DNA methylation patterns in the human genome. These proteins, called *de novo* DNA methyltransferases (DNMTs), comprise two active enzymes, DNMT3A and DNMT3B, and one stimulatory factor, DNMT3L. Our study clearly establishes that DNMT3A and DNMT3B do not methylate DNA at random but rather that they show strong and distinct preferences for their target sites *in vivo*. These preferences lead to the deposition of unique and reproducible patterns of methylation and may have contributed to shaping segments of the human genome. In contrast, we show that DNMT3L stimulates DNA methylation mostly at sites that are poorly methylated on their own, thus leading to patterns that are more uniform. This modulation is proposed to result from DNMT3L anchoring the DNA methylation machinery onto chromatin, the physiological form under which DNA exists in our cells. This study furthers our understanding of how genomic DNA methylation patterns are established *in vivo*.

## Introduction

Cytosine DNA methylation, which is primarily focused at symmetrical CpG sites in mammalian cells, represents a critical epigenetic mark broadly associated with silent genomic regions. Repeated DNA elements such as dispersed transposon-derived repeats or heterochromatin-associated pericentric satellite repeats are heavily methylated, highlighting the primordial role of DNA methylation as a genome defense mechanism [1]. Cytosine DNA methylation is also essential for development [2], [3] and contributes to the regulation of gene expression through differentiation [4]–[6]. Once DNA methylation is established on both DNA strands at a CpG site, it is propagated with high fidelity at each cell division [7]. This stems directly from the fact that hemi-methylated CpG sites, a key intermediate generated by replicating through a fully methylated CpG sequence, are preferentially methylated back to a fully methylated state by the maintenance DNA methyltransferase DNMT1 [8], [9] in association with other interacting factors such as UHRF1 [10], [11]. Thus, DNA methylation profiles represent an important form of epigenetic memory.

Much progress has been made in recent years in our understanding of how cytosine DNA methylation patterns are established during development. This *de novo* methylation function is assigned primarily to the DNMT3 family of DNA methyltransferases (DNMTs) [9]. This family comprises the two active DNMT3A and DNMT3B enzymes, which are highly expressed at specific developmental times in germ cells and during early development, and mediate genome-wide acquisition of DNA methylation. *In vivo*, DNMT3A and DNMT3B possess both overlapping and specific targets. DNMT3A is particularly required for the methylation of imprinted genes and dispersed repeated elements, such as retrotransposons, while DNMT3B specializes in the methylation of pericentric satellite repeats [3], [12]–[14]. The DNMT3L protein, a non-catalytic accessory factor, also serves as an important structural and functional accessory factor for almost all types of *de novo* DNA methylation, particularly in germ cells [15], [16], [17].

The mechanisms by which specific DNA methylation patterns are instructed by DNMT3A, DNMT3B and DNMT3L in the mammalian genome are currently unclear. Multiple studies have indicated that chromatin composition and modification are key in setting the accessibility of certain genomic loci to the DNA methylation machinery. For instance, DNMT3L was proposed to focus DNA methylation away from CpG island promoter regions by discriminating against binding to nucleosomes marked by trimethylation at lysine 4 on histone H3 [5], [18], [19]. Likewise, recent data indicate that the presence of the H2A.Z variant is protective against DNA methylation in the model plant organism *Arabidopsis thaliana* [20]. Other mechanisms, such as recruitment of the *de novo* methylation machinery by direct association with various DNA binding proteins [21]–[23] or possibly by small non-coding RNAs [24], [25], are also likely to operate.

In this study we addressed the possibility that the human DNMT3A and DNMT3B enzymes possess an intrinsic preference for certain DNA sequences flanking their target CpG site. This notion is supported by the concept that the catalytic domains of mammalian DNMTs have evolved from bacterial methyltransferases, many of which are sequence-specific modifying enzymes [9]. Moreover, recent genome-wide bisulfite sequencing efforts have revealed clear local sequence preferences for cytosine methylation in *A. thaliana*, an organism that harbors two *de novo* DNA methyltransferases distantly related to the mammalian DNMT3A and DNMT3B enzymes [26], [27]. Finally, biochemical approaches using purified DNMTs in *in vitro* methylation reactions on naked DNA templates have also hinted at the fact that the mammalian DNMT3A and DNMT3B enzymes might possess an intrinsic flanking sequence preference [28], [29]. However, no specific consensus could be readily derived from these studies.

Here we have used a well-described episomal DNA methylation assay [30], [31] to determine whether the full-length human DNMT3A and DNMT3B enzymes show any flanking sequence preference *in vivo*. For this, we used human HEK293c18 cells, which show little to no endogenous *de novo* methylation activity, and co-transfected target episome DNA together with expression vectors for DNMT3A and DNMT3B in the presence or absence of the DNMT3L protein. The resulting methylation patterns were then determined at various test regions on the episome using bisulfite methylation sequencing and further validated at two additional targets. Our data clearly indicate that DNMT3A and DNMT3B show significant and distinct flanking sequence preferences and reveal a novel and unexpected role of DNMT3L in modulating DNA methylation pattern formation.

## Results

### An assay to measure *de novo* methylation preferences *in vivo*

The episomal methylation assay [30] offers a powerful and versatile tool for measuring DNA methylation in human cells in culture. This assay revolves around the use of unmethylated, stably replicating minichromosomes that are transfected in HEK293c18 cells together with expression vectors for the DNMT(s) of interest. Conveniently, HEK293c18 cells show little if any endogenous *de novo* methylation but efficiently carry out maintenance methylation [30]. Thus, the *de novo* methylation patterns established on these target episomes by exogenous DNMT(s) expressed in these cells are stably maintained for prolonged periods of time. Methylation patterns can then be detected at nucleotide resolution using bisulfite methylation sequencing [32]. Here we analyzed DNA methylation at four distinct regions carried on episomal constructs. All regions fit a strict operational definition for a CpG island, namely GC content >55% and a ratio of observed versus expected CpG sites ratio >0.8 [33]. Focusing on CpG-rich regions enabled us to maximize the range of sequence flanks analyzed; altogether 271 distinct CpG sites were studied.

To validate these episomal constructs as a good tool for determining the intrinsic sequence preference of the human DNMT3A and DNMT3B enzymes, we wanted to ensure that *de novo* methylation could not trigger DNMT1-mediated “spreading” effects around pre-methylated sites [34]. If spreading were to occur, it would diminish our ability to discern true *de novo* activity by the *de novo* enzymes from DNMT1-mediated activity. To test this, we methylated the pFC19 episome *in vitro* with the HhaI DNA methyltransferase and transfected the DNA in HEK293c18 cells. After 7 days, the episomal DNA was recovered by Hirt harvest and DNA methylation patterns were determined using bisulfite methylation sequencing. The methylation pattern on the input DNA prior to transfection was also determined for comparison. The data clearly show that methylation patterns were faithfully maintained without significant modification from the initial pattern (data not shown). Thus, DNMT1 does not appear to lead to “spreading” effects in this sytem. We also transfected the pre-methylated episomes together with expression vectors for DNMT3A or DNMT3B to determine if pre-methylated sites might attract the *de novo* enzymes to their immediate vicinity (“seeding” or “clustering” effects). We determined the methylation patterns and compared them to the patterns obtained with unmethylated episomes and observed no significant changes (data not shown). This indicates that pre-existing CpG methylation does not stimulate DNMT3A or DNMT3B activity, in agreement with another independent study [35].

Finally, since our episomes are generated in *E. coli* and therefore carry Dam methylation (N6 Adenine methylation at GATC sequences) and Dcm methylation (C5 methylation at the internal cytosine in CC^A^/_T_GG sequences), we verified that such non-CpG methylation marks do not modify the CpG methylation patterns laid by DNMT3A or DNMT3B. For this, episome DNA was extracted from *dam^−^*, *dcm^−^*, and *dam^−^ dcm^−^ E. coli* strains and used for transfections together with expression vectors for DNMT3A or DNMT3B. The distribution of methylated sites, as judged by Southern blots after digestion with methyl-sensitive restriction enzymes, did not detectably vary (data not shown), indicating that pre-existing non-CpG methylation does not influence the activity of the *de novo* enzymes. Similar observations have been reported [35]. The presence of 5-methylcytosine at Dcm sites, however, provided us with the ability to track whether a particular DNA strand has been newly synthesized in human cells upon replication of the episome or corresponds to an “old” bacterial DNA strand that was transfected in. Indeed, non-CpG methylation is not maintained in human cells and newly synthesized DNA strands were consistently devoid of dcm methylation.

### DNMT3A shows a significant flanking sequence preference for methylating DNA *in vivo*

**Figure 1:**
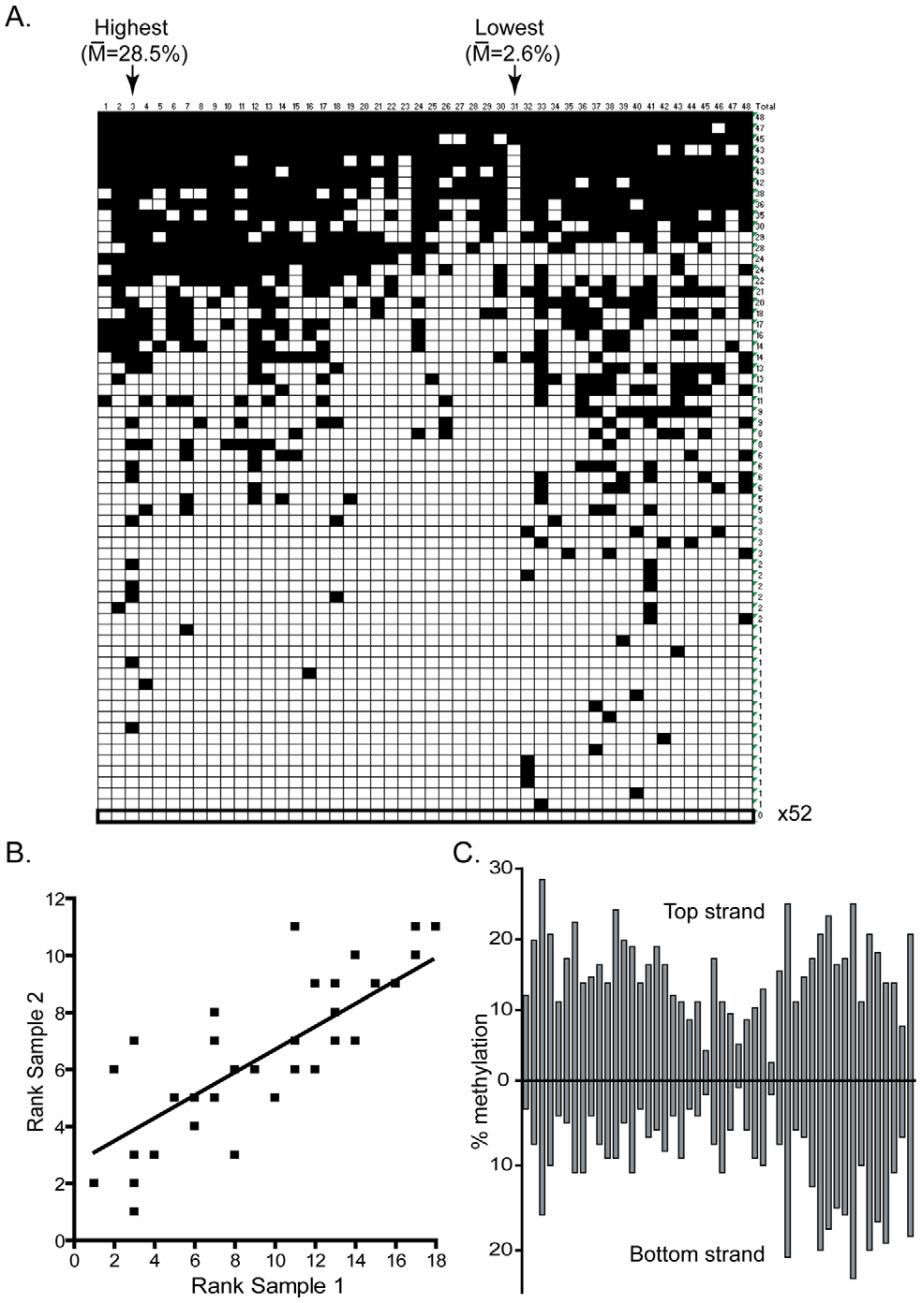
DNMT3A methylation patterns are characterized by reproducible and symmetrical high and low efficiency methylation sites. **A.** This panel represents the DNA methylation patterns deposited by DNMT3A at the pBR top strand region as determined by bisulfite methylation sequencing. Each horizontal line corresponds to an independent DNA molecule while each vertical line corresponds to one of the 48 CpG sites in this region. Black squares indicate CpG methylation while white squares represent absence of methylation. The arrows indicate the two sites with the greatest and least methylation frequencies, respectively, along with their corresponding methylation frequencies. **B.** Two independent *in vivo* samples were analyzed for their DNA methylation patterns and the CpG sites were ranked according to their methylation efficiency. The ranks observed at each of the 48 CpG sites were then plotted against each other in one sample versus another. Overall, a good correlation exists between methylation ranks, indicating that the methylation patterns for DNMT3A are reproducible. **C.** The individual methylation efficiency at each CpG site along the pBR322 region are plotted for both top and bottom DNA strands. The patterns observed are clearly symmetrical across DNA strands.

Methylation of the target minichromosome by DNMT3A was analyzed by bisulfite methylation sequencing at two test regions (Hygro and pBR – See Material and Methods) on both DNA strands. In total, 20,352 CpG sites were sequenced for the DNMT3A sample, representing over a 100-fold coverage of all available sites. For most regions, the methylation at various CpG sites was not uniform but rather showed clear evidence for high and low methylation sites, as evidenced for the pBR Top strand region (Figure 1A). While the overall methylation efficiency on that strand was 15.3%, the methylation was not evenly distributed between all 48 CpG sites, leading to the formation of a clear pattern characterized by high and low efficiency sites. Site # 3, for instance, was methylated with an efficiency of 28.5% while site # 31 was methylated with an 11-fold lower efficiency of 2.6%. The presence of hotspots and coldspots for DNA methylation and the overall pattern of DNA methylation resulting from DNMT3A activity was reproducible in a completely independent biological replicate. For example, Figure 1B shows that when CpG sites were ranked according to their individual methylation efficiencies in each sample, a clear positive correlation is observed between the two independent replicates. This reflects the fact that top methylation sites (low ranks) remain highly methylated in both samples while bottom methylation sites (high ranks) show consistently poor methylation in both samples. Indeed, 6 out of the 10 top CpG sites in sample 1 also belonged to the top 10 sites in sample 2. Conversely, 7 out of the 10 bottom CpG sites in sample 1 also belonged to the bottom 10 sites in sample 2 for that particular region. Similar observations of consistent high and low efficiency sites were made at the Hygro region with individual variation in methylation efficiencies of up to 6-fold (data not shown).

Consistent with the presence of endogenous maintenance DNA replication in HEK293 cells, the methylation patterns observed at each region were mostly symmetrical between the two DNA strands. As shown in Figure 1C, a strong correlation of methylation efficiencies is observed across the two strands of the pBR region. This observation has implications for our ability to properly identify and score the flanking sequences of high and low methylation sites. Indeed, the presence of a methylation hotspot on one strand should lead to the observation of a high methylation site on the other strand due to maintenance methylation. Therefore, some sites that appear as methylation hotspots may not directly correspond to a hotspot but may be located across a hotspot on the other strand. In contrast, this predicts that methylation coldspots should correspond to low methylation efficiency sites on both DNA strands, as observed.

**Figure 2:**
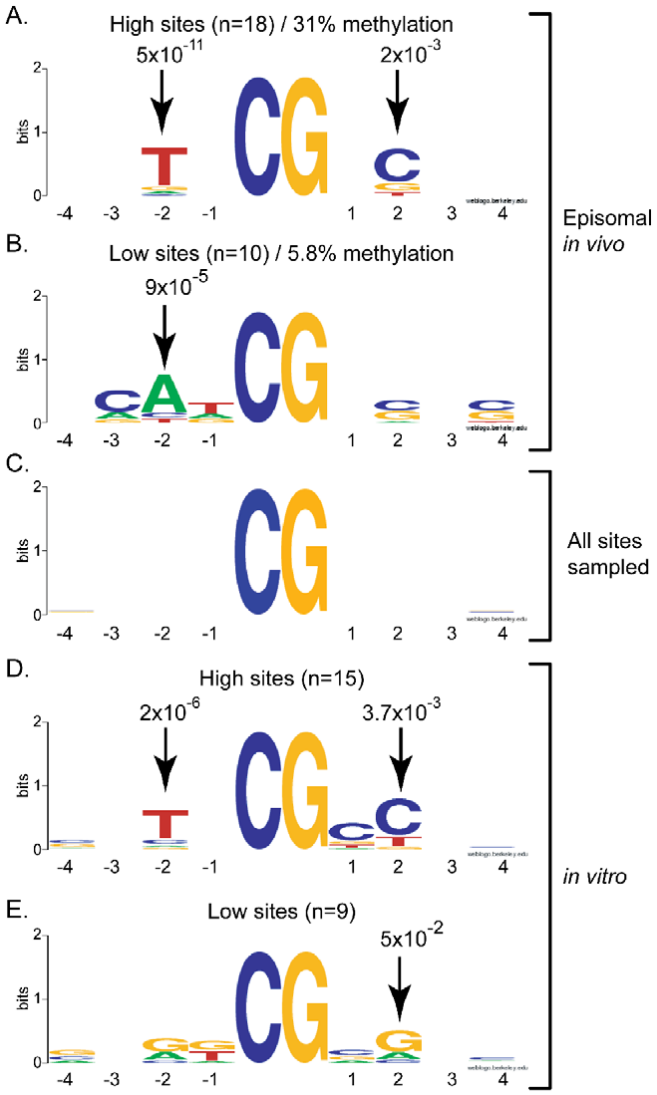
DNMT3A shows selectivity for residues flanking the target CpG site at positions −2 and +2. The sequences flanking the most (panels A, D) and least (panels B, E) methylated sites for DNMT3A were extracted and aligned as described in the text. Residues that were enriched at any position in the 4 base-pairs surrounding the target CpG site on each side (x-axis) are represented in the Logos format whereby sequence enrichment at each position is indicated by the size of each letter in bits (y-axis; 2 reflects perfect conservation while 0 reflects a random distribution). The corresponding P-values measuring the enrichment of a given residue over the input DNA sequence are indicated for positions showing significant preference. Information was derived either from our episomal assay *in vivo* (panels A and B) or using purified DNMT3A complexes *in vitro* (panels D and E). Panel C corresponds to the entire set of CpG sites analyzed here – these sites showed no intrinsic sequence enrichment.

To determine if the high and low methylation sites observed as a result of DNMT3A activity could be explained by a potential flanking sequence preference, we focused on the sequences flanking the 10% most methylated CpG sites and the 10% least methylated CpG sites at the pBR and Hygro regions. For this, all CpG sites within each region were ranked according to their respective methylation efficiencies and the flanking sequences extracted on each side of the target CpG site. The sequences were then systematically aligned with each other either in direct or reverse-complement orientation to identify regions of similarity in each class. In the case of DNMT3A, it rapidly became apparent that methylation hotspots were likely to share a T residue at the −2 position and a C residue at the +2 position from the target CpG sites (Figure 2A). The over-representation of the T and C residues at these positions over the average sequence composition of the regions under study was statistically significant with p-values of 5×10^−11^ and 2×10^−3^, respectively. Interestingly, low efficiency sites, which on average were methylated at a 5.3-fold reduced efficiency compared to high sites, also showed statistically significant enrichment for adenine residues at position −2 (Figure 2B), indicating that this position is particularly important for discriminating between a good and bad flank. Examination of up to 12 positions on each side of the target CpG site revealed that positions −2 and +2 were the only positions to show strong statistical significance (data not shown). Similar results were observed when the shorter DNMT3A2 isoform [36] was used as judged from the strong correlation of methylation efficiencies at individual CpG sites (Figure S1). As expected, no enrichment was observed when the entire set of CpG sites analyzed here was aligned (Figure 2C).

### DNMT3A shows strong intrinsic flanking sequence preference *in vitro* consistent with *in vivo* observations

To determine if the flanking sequence preference observed for DNMT3A in HEK293 cells corresponds to an intrinsic enzymatic preference, we used the purified catalytic domain of DNMT3A and performed *in vitro* DNA methylation reactions. The resulting methylation at the pBR region was analyzed by bisulfite sequencing on both DNA strands. As observed *in vivo*, the methylation patterns indicated clear preference for some sites over others with a 15-fold maximal range between high and low sites (data not shown). When the sequences flanking the 15% top and bottom sites were extracted and aligned, a pattern similar to the one observed *in vivo* emerged. Top methylated sites (average methylation efficiency 88.2%) tended to carry a T at position −2 and a C at position +2 (Figure 2D). By contrast, the least methylated sites (average methylation efficiency 13.4%) showed an enrichment for A or G residues at the −2 position and a G at position +2 (Figure 2E). Thus, the sequence composition at the −2 and +2 positions appears critical in selecting for a good or bad flank for DNMT3A *in vivo* and *in vitro*. Moreover, since *in vitro* methylation patterns are not compounded with any maintenance methylation, this indicates that the DNMT3A preference was properly assigned *in vivo* and that this preference represents an intrinsic property of the catalytic site of the enzyme. Interestingly, nearly 25% of all methylated cytosines observed *in vitro* were found in CpA and CpT contexts, clearly showing that DNMT3A is capable of non-CpG methylation activity, in agreement with previous studies [37], [38]. Highly methylated non-CpG sites also showed an enrichement for a T at the −2 position (data not shown). The high preponderance of non-CpG methylation *in vitro* is in contrast to the situation observed *in vivo* as episomal substrates showed little to no non-CpG methylation (data not shown). This is likely due to the fact that methylated non-CpG sites are not maintained upon replication by DNMT1.

### *In vivo* methylation by DNMT3B reveals a significant flanking sequence preference distinct from DNMT3A

**Figure 3:**
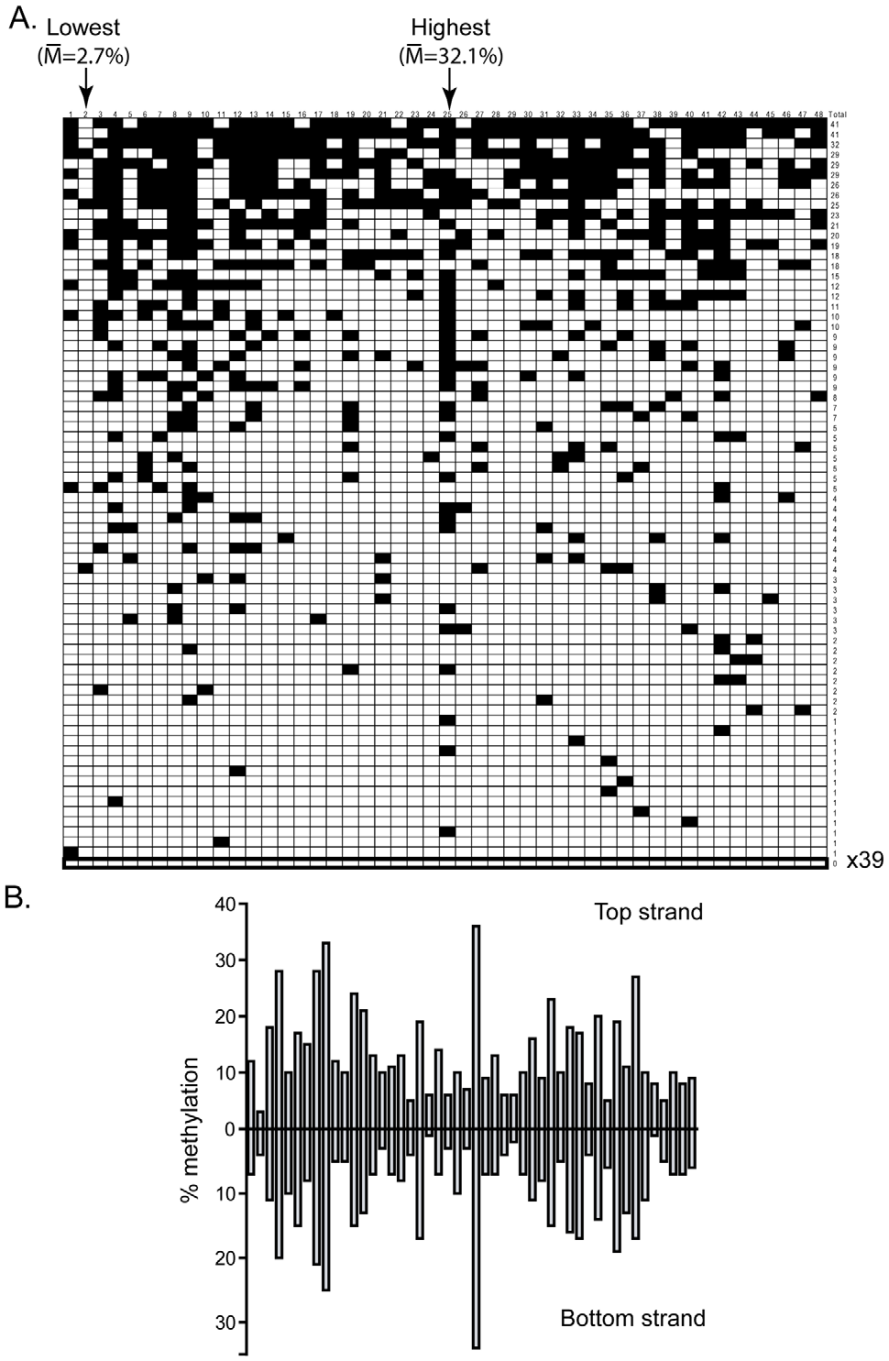
DNMT3B shows significant sequence preference distinct from DNMT3A. **A.** This panel represents the DNA methylation patterns deposited by DNMT3B along the pBR top strand region. Symbols are as described above. Note that the overall pattern of DNA methylation is strikingly different from the one observed for DNMT3A (Figure 1A). The most and least methylated sites in this region are indicated with an arrow along with their respective methylation frequency. **B.** The individual methylation efficiency at each CpG site along the pBR322 region are plotted for both top and bottom DNA strands. As observed for DNMT3A, the patterns are clearly symmetrical across DNA strands.

The DNMT3B *in* vivo methylation patterns were next analyzed in the same manner as DNMT3A. In total 20,203 CpG sites were sequenced for the DNMT3B1 sample, again representing over a 100-fold coverage. Detailed inspection of the patterns deposited by DNMT3B at the pBR and Hygro regions revealed that the patterns showed distinct and reproducible high and low frequency methylation sites (Figure 3A). Importantly, the DNMT3B patterns were different than those for DNMT3A with distinct CpG positions corresponding to hotspots and coldspots (compare Figure 3A to Figure 1A). Compared to DNMT3A, DNMT3B showed an even greater discrimination between high and low sites: a 34-fold difference was observed between the highest (29.3% methylation efficiency) and lowest (0.86%) sites on the pBR bottom strand. This indicates that, compared to DNMT3A, DNMT3B preferentially methylates, and avoids, different flanking sequences. As observed for DNMT3A, the overall DNA methylation patterns were symmetrical across both DNA strands (Figure 3B).

**Figure 4:**
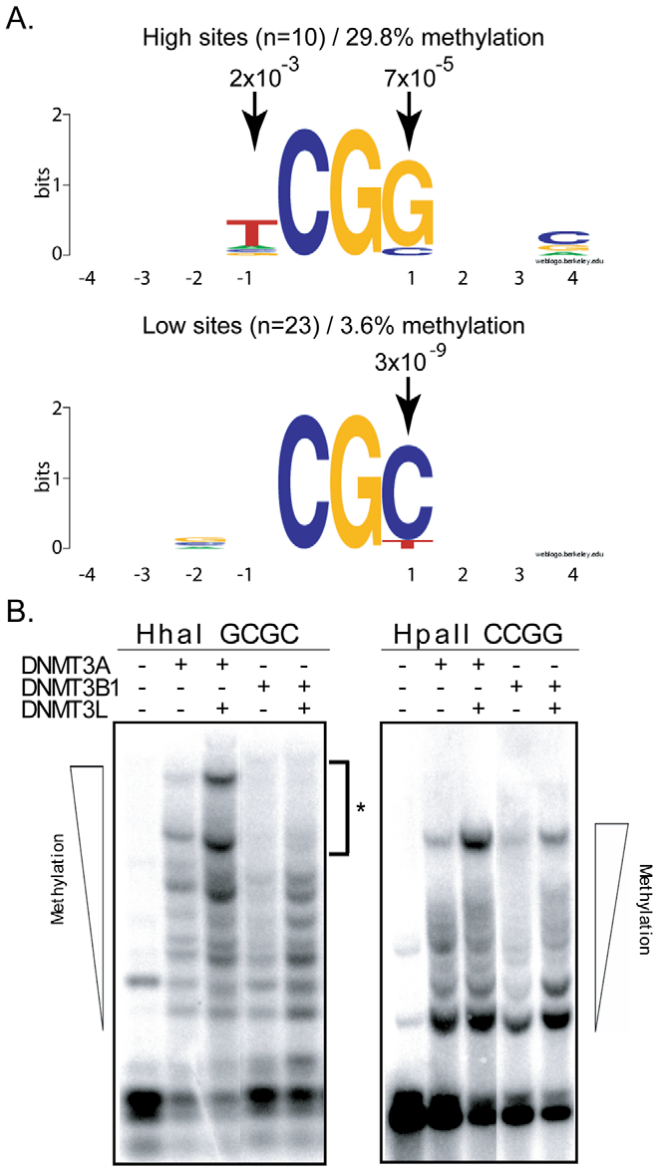
DNMT3B shows selectivity for residues flanking the target CpG site at positions −1 and +1. **A.** The flanking sequence preference for DNMT3B was analyzed from the 10% top (left side) and 10% bottom (right) methylation sites, as described in the text. Conservation of particular residues at each 4 positions flanking the target CpG site is represented as Logos diagrams. **B.** Methylation-sensitive restriction enzymes confirm sequencing data. Methylation sensitive restriction enzyme digestion of DNMT3A and DNMT3B samples followed by Southern blotting reveals the methylation status at HhaI and HpaII sites. For DNMT3B, HhaI sites (5′-GCGC-3′) are predicted to correspond to low efficiency sites. By contrast, HpaII sites (5′-CCGG-3′) are predicted to correspond to high efficiency sites. As shown here, even in the presence of the stimulatory factor DNMT3L, DNMT3B cannot catalyze the complete methylation of the target episomal DNA at HhaI sites (left panel – asterisk). By contrast, DNMT3B can promote complete methylation at HpaII sites (right panel). Also as predicted, DNMT3A shows similar methylation at both sites.

To extract the flanking sequence preference for DNMT3B, we focused on the 10% most methylated and the 10% least methylated CpG sites at the two test regions and aligned these sites with respect to the central CpG site. DNMT3B hotspots revealed an enrichment for a T residue at position −1 and a G residue at position +1 with p-values of 2×10^−3^ and 7×10^−5^, respectively. Coldspots, by contrast, showed a strong enrichment for a C residue at position +1 with a p-value of 1×10^−8^ (Figure 4A). Thus, unlike DNMT3A, which discriminates between flanks by the composition of the −2 and +2 flanks, DNMT3B appears to respond mostly to the sequence composition at the −1 and +1 positions.

To validate these observations, we sought to determine whether methylation by DNMT3B would be more prevalent at 5′-CCGG-3′sites, which are recognized by the methylation-sensitive HpaII enzyme, than at 5′-GCGC-3′ sites, which are recognized by the methylation-sensitive HhaI enzyme. Our observation that a G at the +1 position is a hallmark of high efficiency sites while a C in this position is highly enriched in low efficiency sites predicts this outcome. Upon cleavage of our Hirt harvest episomal vector with HpaII and HhaI, the resulting DNA fragments were separated by gel electrophoresis and the DNA methylation patterns were revealed by Southern blotting. As shown in Figure 4B, high molecular weight bands corresponding to the methylation of almost all available sites can be readily observed upon digestion with HpaII. By contrast, we reproducibly failed to detect such high mobility species upon cleavage by HhaI even in the presence of the stimulatory factor DNMT3L, indicating that HhaI sites remained available for cleavage and therefore were unmethylated. This indicates that HhaI sites, which carry a C at the +1 position, are poorly methylated in contrast to HpaII sites, which carry a G at the +1 position, in agreement with our sequencing data. As also predicted by our analysis, methylation of episomal DNA by DNMT3A did not lead to any measurable distinction in the cleavage efficiency by HpaII or HhaI (Figure 4B). These observations therefore validate our sequencing data using an independent method and indicate that most of the variation in methylation efficiencies for DNMT3B is indeed captured by the −1 and +1 positions from the target CpG site since HhaI is insensitive to all other positions. We also independently examined DNA methylation activity for the active DNMT3B2 splice isoform and observed that the flanking sequence preference for DNMT3B2 was essentially unchanged compared to the full-length DNMT3B1 protein (Figure S1).

### Validation of sequence preference at mammalian CpG islands

While the test regions used so far correspond broadly to CpG islands, we wished to validate the preferences observed using sequences directly of human origin. For this, two human CpG islands were cloned into episomal constructs and used as sequence targets.

The first region analyzed corresponded to a 539 bp portion of the imprinted and maternally methylated *SNRPN* CpG island carrying 37 CpG sites. This region, while GC-rich overall, shows a strong strand asymmetry in the distribution of guanine and cytosines outside of CpG sites such that one strand is highly G-rich and the other highly C-rich, a property referred to as GC skew. Interestingly, the overall methylation efficiencies of the two strands were significantly different (DNMT3A: C-rich 18.5%/G-rich 31.5%; DNMT3B: C-rich 9.7%/G-rich 21.7%) despite the presence of efficient maintenance methylation at other regions tested on the same episomes. This suggests that the *de novo* enzymes are recruited preferentially to the G-rich strand and/or that the maintenance machinery has difficulty maintaining the methylation patterns at these regions (an intrinsic bias in our ability to detect highly methylated C-rich strands is unlikely as such molecules can be efficiently recovered upon *in vitro* methylation; data not shown). Inspection of the patterns deposited on both strands revealed that the G-rich strand was also more uniformly methylated (maximal fold difference between the most and least methylated sites: 2.6 and 3-fold for DNMT3A and DNMT3B, respectively) than the C-rich strand (maximal fold difference between the most and least methylated sites: 5.6 and 23-fold for DNMT3A and DNMT3B, respectively).

When focusing on the C-rich strands, which show variation in the patterns, we noted that for DNMT3A (81 independent molecules were analyzed), two of the the top three sites (average methylation efficiency 33%) displayed a T at position −2 and two displayed a C at position +2. By contrast, the bottom five sites (average methylation efficiency 8.9%) were flanked by either an A at position −2 (three cases out if five) or a G at position +2 (2 cases out of five) (Figure S2). Thus, the variation in methylation efficiencies observed at the *SNRPN* region for DNMT3A recapitulated the preference observed at our test regions. Likewise for DNMT3B (88 independent molecules were analyzed), six out of the seven bottom sites (average methylation efficiency 2.6%) carried a C at position +1, while only one out of the four top sites (average methylation efficiency 20.9%) carried a C at this position (Figure S2).This is again compatible with the observations reported above for test regions.

**Figure 5:**
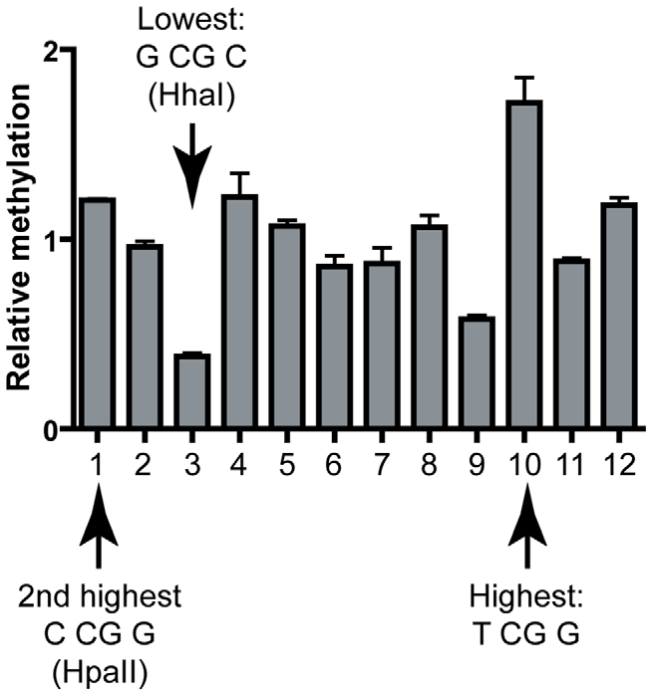
Validation of the DNMT3B sequence preference at the human *TIMELESS* CpG island. The methylation frequencies observed at each CpG site are shown relative to the average frequency observed for the region (*i.e.* a value of 2 would indicate that the frequency at a given site is two-fold above average). Each bar represents the average of three independent experiments; error bars indicate standard deviation. Hotspots and coldspots are indicated and are as expected from the sequence preferences derived from test regions.

We also analyzed DNA methylation patterns at the CpG island from the *TIMELESS* promoter which was cloned in our episomal construct in place of the *SNRPN* region. In this case, the methylation of 12 CpG sites were investigated by quantitative methylation pyrosequencing. Upon expression of DNMT3A, methylation patterns were remarkably consistent over three independent experiments and showed only little variation: the maximal fold difference in methylation efficiencies between the most and least methylated sites was 1.8-fold (data not shown). Thus little information could be derived. In the case of DNMT3B, however, a highly reproducible pattern could also be detected and the most and least methylated sites showed an average 4.4-fold difference in methylation efficiencies (Figure 5). Strikingly, site #10, the most methylated site in this sequence corresponded to the predicted hotspot TCGG, while the second most methylated site (Site #1) corresponded to an HpaII site (CCGG), also predicted to represent a hotspot. Furthermore, the least methylated site, site #3, mapped onto the predicted coldspot GCGC (HhaI site), while the second least methylated site (Site #9) mapped to the palindromic CACGTG sequence. Sites with intermediate methylation efficiencies did not fit to either consensus motifs for predicted high and low sites. Altogether, this suggests that the sequence preferences derived from test regions do apply broadly to various sequences regardless of their origin.

### Evidence for altered distribution of hotspot and coldspot sequences in human CpG islands

In the euchromatic portion of the human genome, the CpG dinucleotide is mostly confined to CpG islands. Within these loci, one can distinguish between two classes of islands: “specific” CpG islands serve as promoters and are seldom methylated; “weak” CpG islands are located in the bodies of genes or in intergenic regions and are often methylated and silent [39]. It is tempting to speculate that the relative unmethylated state of “specific” CpG islands might be explained at least in part by an under-representation of DNA methylation hotspots and/or perhaps an over-representation of DNA methylation coldspots. In contrast, one might expect that weak CpG islands might show little evidence for selection of particular sequence motifs.

**Figure 6:**
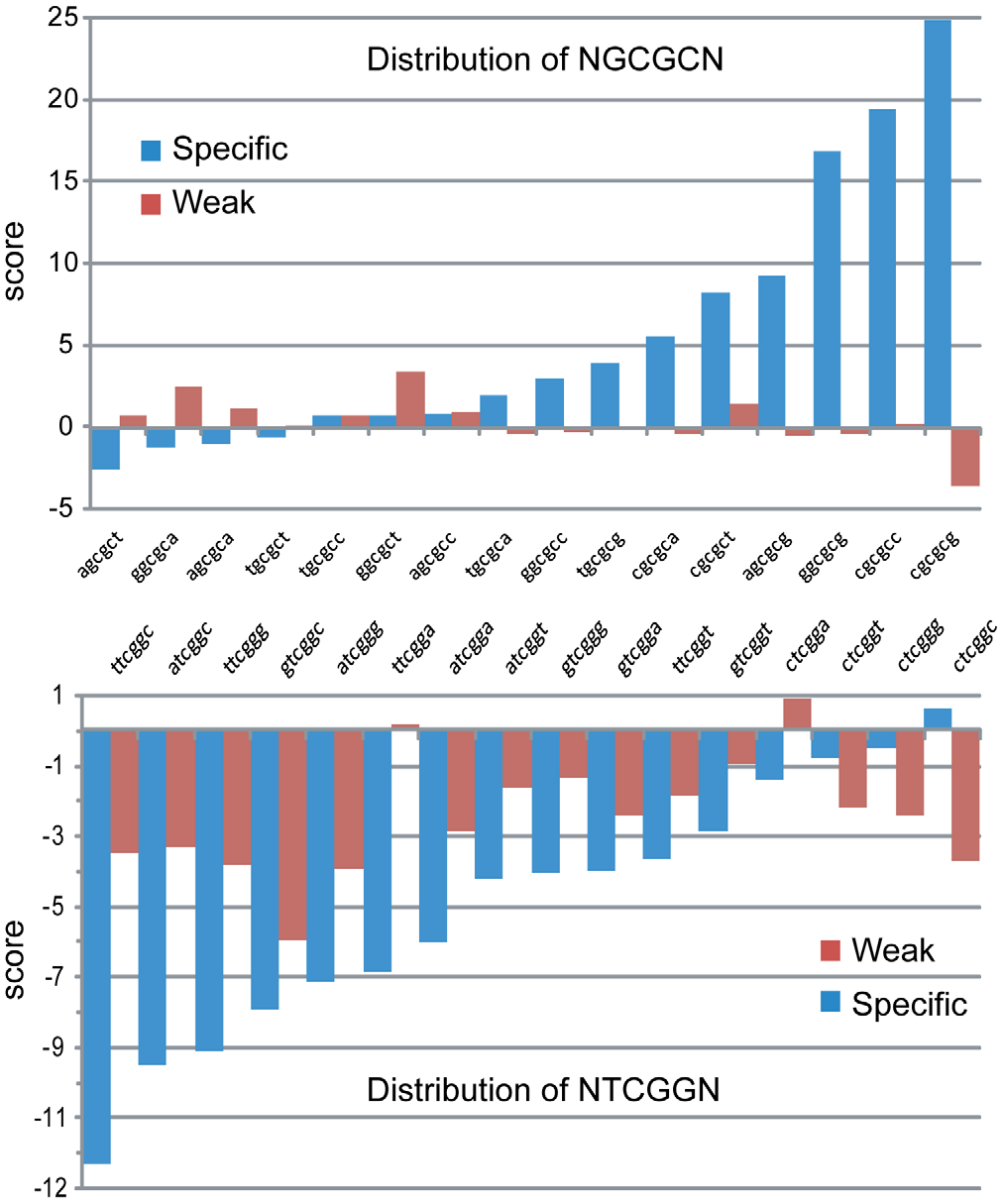
Human CpG islands show evidence of selection at methylation signature motifs. The distribution of predicted DNMT3B methylation coldspots (5′-NGCGCN-3′, top) and hotspots (5′-NTCGGN-3′, bottom) was analyzed in two datasets corresponding to all “specific” and all “weak” CpG islands from human chromosome 1, respectively. The score on the y-axis represents the exceptionality of the frequency of a given motif. A positive score indicates an over-representation of a motif in a given model of the sequence (the M1 model, which computes expected motifs frequencies based on mono- and di-nucleotide frequencies was used here). A negative score indicates under-representation. A higher absolute score value indicates a greater statistical significance (a score of 5 corresponds to a p-value of ∼10^−6^, which starts to be considered significant; a score of 10 corresponds to a p-value of ∼10^−23^).

To evaluate this possibility, we used the R'MES statistical package, which uses Markovian models to evaluate the exceptionality of motif (or ‘word’) frequency in DNA. When looking at 6-letter words under the M1 model, which analyzes a given DNA sequence based on mono- and di-nucleotide frequencies, we first determined that words fitting the NNCGNN consensus showed a much greater range of over- or under-representation in the “specific” set compared to the “weak” set of CpG islands (data not shown). Interestingly, we determined that NGCGCN sites, which are predicted to represent DNMT3B methylation coldspots, showed significant over-representation in the set of “specific” CpG islands, but not in “weak” CpG islands in an M1 model (Figure 6). By contrast, we determined that NTCGGN sites, which are predicted to represent DNMT3B DNA methylation hotspots, tended to be under-represented in the set of “specific” islands (Figure 6). This under-representation was much less visible in the “weak” CpG islands. Interestingly, the most under-represented NNCGNN word in the set of “specific” CpG islands was 5′-TTCGGC-3′, a predicted sequence hotspot for both DNMT3A and DNMT3B. For this word, less than half of the expected occurences could be counted (249 out of 530, respectively), which is associated with a strong statistical significance (p-value<10^−34^). However, we could not detect further evidence for enrichment or depletion based on the −2 and +2 position of NNCGNN sites. This analysis suggests that the intrinsic flanking preference of DNMT3B, and to a lesser extent DNMT3A, might have contributed to shaping promoter CpG island sequence composition in the human genome.

### DNMT3L focuses *de novo* methylation on properly chromatinized DNA templates

**Figure 7:**
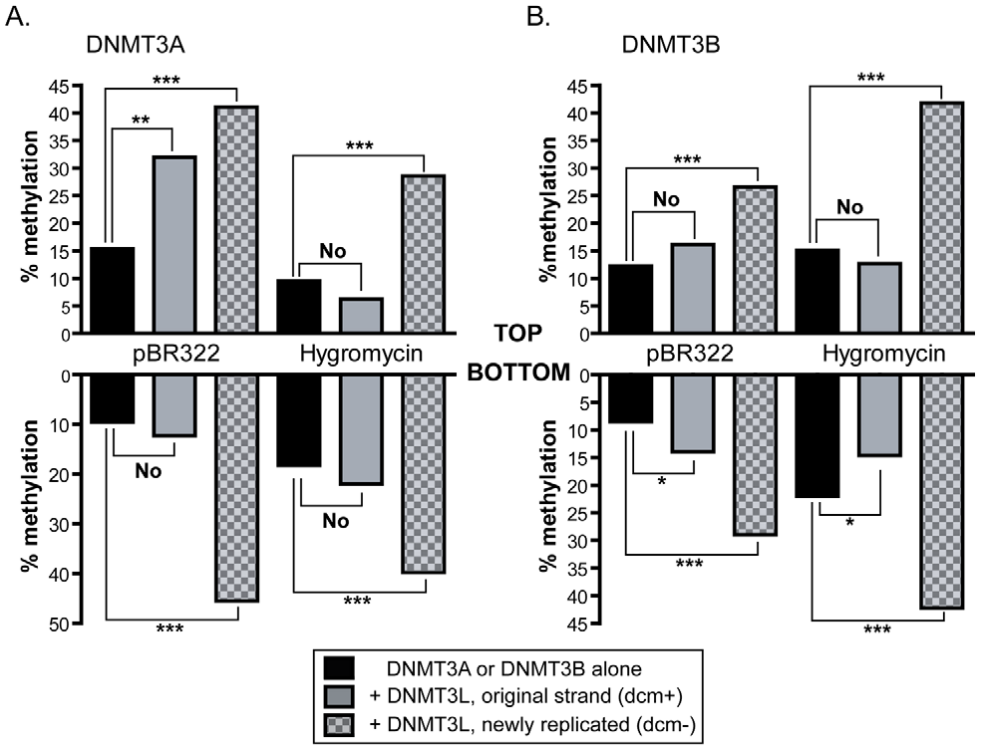
Stimulation by DNMT3L is observed primarily on newly replicated DNA strands. The overall methylation efficiencies (in percentages) observed on both strands at the pBR322 and Hygro regions are indicated in black bars for DNMT3A (panel A) and DNMT3B (panel B). The number of independent DNA molecules taken into account to calculate the average methylation efficiencies varied from 36 molecules to 120 per sample. In the presence of DNMT3L, the data were broken down between dcm+ (grey bars) and dcm− (checkered grey bars) DNA strands, as indicated. Whether the stimulation afforded by DNMT3L was significant or not was determined by a one tailed Student t-test and is indicated graphically (‘no’, not significant; ‘***’ P-value<0.001; ‘**’ P-value between 0.001 and 0.01; ‘*’ P-value between 0.01 and 0.05). While DNMT3L-mediated stimulation of DNA methylation is readily observed at all tested regions on newly synthesized DNA molecules (dcm−), it is not observed in the majority of cases for dcm+ molecules.

Having determined the flanking sequence preference of DNMT3A and DNMT3B on their own, we then examined the effect of the DNMT3L protein on the formation of DNA methylation patterns. DNMT3L, as described previously, is a potent stimulatory factor for *de novo* methylation. However, at first inspection, our data revealed only a moderate 1.5 to 2-fold stimulation of DNA methylation by DNMT3L, which was somewhat lower than previously observed *in vivo* [40], [41]. Upon closer examination of sequencing data, we noticed a significant difference between DNA strands that had been newly synthesized inside HEK293 cells (characterized by the fact that they lost the original dcm bacterial methylation marks; these strands are thereafter referred to as dcm−) and the original DNA strands that were transfected (these strands carry the dcm bacterial marks and are referred to as dcm+). Namely, we observed that newly replicated DNA strands (dcm−) showed much stronger levels of stimulation by DNMT3L than dcm+ strands, which for most regions examined showed no significant stimulation by DNMT3L (Figure 7). Hence, the average stimulation on dcm+ strands at the pBR322 and Hygro regions for DNMT3A was 1.3-fold, while it was 3.2-fold on dcm− strands. A similar observation was made for DNMT3B. Interestingly, this distinction between dcm− and dcm+ DNA strands only applied when DNMT3L was present; the methylation efficiencies of these strands were very similar when DNMT3A or DNMT3B were considered on their own (data not shown).

This strongly suggests that the effect of DNMT3L is mostly felt on newly replicated DNA strands. There are two hypotheses that could explain this observation. One is that stimulation of DNA methylation by DNMT3L might be mechanistically coupled to DNA replication. The other is that DNMT3L might focus DNA methylation towards well-chromatinized DNA templates, thus biasing the DNA methylation machinery towards newly replicated episomal DNA molecules that have acquired chromatin with fork passage. No evidence exists so far for a coupling between *de novo* DNA methylation and DNA replication. On the contrary, DNMT3L clearly promotes *de novo* methylation in non-dividing cell types [42]. Likewise, recent evidence shows that DNMT3L physically binds to chromatin [18], [19].

To distinguish between these two hypotheses, we devised an experiment that allowed us to track DNMT3L stimulation as a function of replication on fully chromatinized episomes. Episomes were transfected in HEK293c18 cells and allowed to propagate under selective pressure through multiple rounds of replication encompassing at least 20 cell generations. At this point the episomes are expected to be fully chromatinized and episomal DNA no longer carried detectable dcm methylation (data not shown). To track the replication status of DNA strands we then transiently transfected a vector expressing a MYC-tagged version of the bacterial Dcm methyltransferase carrying a nuclear localization signal to mark DNA strands in a “pulse” of expression. Using Western blots, we determined that the Dcm methyltransferase was efficienctly expressed 24 hours after transfection and remained at high levels in the cells up until ∼5 days post transfections, at which point expression rapidly declined (data not shown).

Two days post-transfection, we extracted the genomic DNA and verified that the DNA had become marked in that it became extensively resistant to cleavage by EcoRII, a restriction enzyme sensitive to dcm methylation (data not shown). In addition, episomal DNA became re-methylated at dcm sites, as determined by bisulfite sequencing (data not shown). This transient pulse of Dcm expression therefore allowed us to re-mark endogenous DNA, enabling us to track the replication status of DNA strands. Seven days after the Dcm expression vector was first transfected, we then introduced expression vectors for DNMT3A in the presence or absence of DNMT3L. The resulting methylation patterns were determined using bisulfite methylation sequencing another seven days after transfection of DNMT vectors. Our prediction was that if DNMT3L function is coupled to DNA replication, then only the newly replicated dcm− molecules should show stimulation. By contrast, if DNMT3L function is independent from DNA replication but is sensitive to the chromatin status of its target molecules, then both dcm− and dcm+ molecules should show stimulation. Consistent with this second hypothesis, DNMT3L triggered a 6.34-fold stimulation of DNA methylation on dcm+ molecules and a 5.76-fold stimulation on dcm− molecules. This indicates that, as expected, DNMT3L stimulation is not dependent upon replication, and suggests that DNMT3L directs DNA methylation to fully chromatinized templates.

### DNMT3L favors the formation of more uniform DNA methylation patterns without altering the intrinsic sequence preferences of DNMT3A and DNMT3B

**Figure 8:**
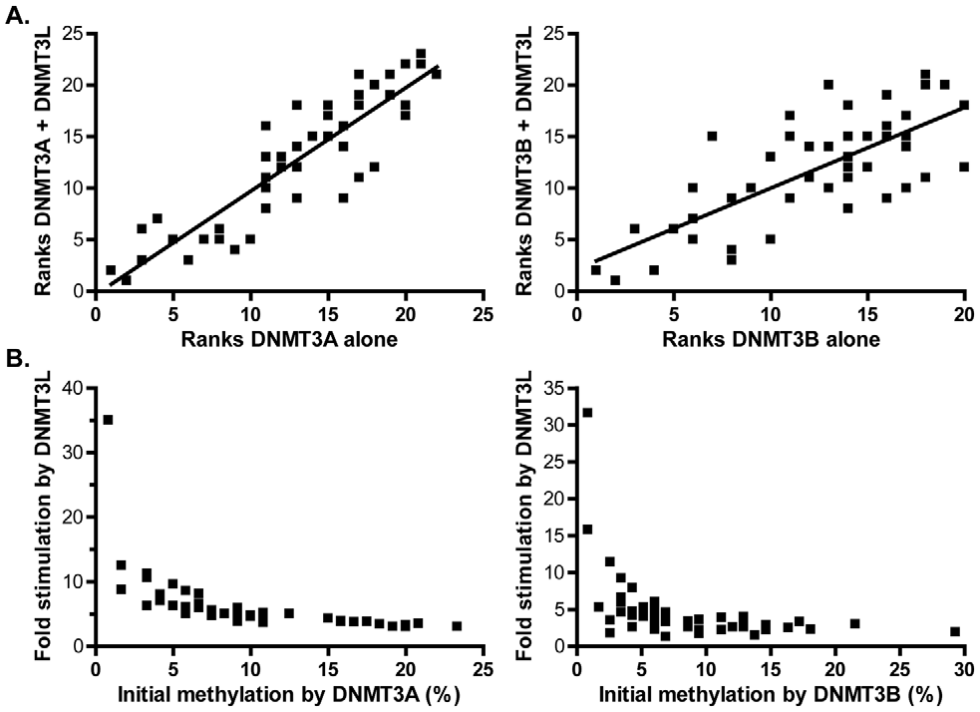
DNMT3L modulates DNA methylation pattern formation by DNMT3A or DNMT3B. **A.** Methylation ranks observed for DNMT3A alone (left) or DNMT3B alone (right) on the pBR322 top strand region are plotted against the corresponding methylation ranks observed when DNMT3L is also introduced (dcm− strands only). A clear correlation is observed, indicating that the overall hierarchy of sites is not changed (r^2^ values are 0.80 for DNMT3A (left) and 0.55 for DNMT3B (right)). **B.** Fold stimulation exerted by DNMT3L at each individual site is plotted as a function of each site's corresponding methylation efficiency in the absence of DNMT3L. The sites with the lowest initial methylation in the absence of DNMT3L clearly show the strongest stimulation, leading to the formation of more uniform DNA methylation patterns.

While it is clear that DNMT3L stimulates the catalytic activity of its partners, it remains to be determined if the stimulation is accompanied by any change in the flanking sequence preference of DNMT3A or DNMT3B. To determine this, we ranked CpG sites according to their individual methylation efficiencies in the presence and absence of DNMT3L and compared the ranks between the two categories. DNMT3L, while it strongly stimulated DNA methylation by DNMT3A on dcm− strands, did not alter the rankings of high and low efficiency sites (Figure 8A left). Similar results were observed for DNMT3B (Figure 8A right). In addition, DNA methylation patterns deposited *in vitro* by the full length DNMT3A2 enzyme in complex with DNMT3L clearly showed evidence of selection for suitable flanks at the −2 position (Figure S3).Thus, it seems likely that DNMT3L does not alter the intrinsic sequence preference of either active enzyme, consistent with the notion that DNMT3L binds to DNA poorly [43].

We noticed, however, that the spread in the individual methylation efficiencies between CpG sites was noticeably reduced in the presence of DNMT3L, thus leading to the establishment of more uniform patterns characterized by long tracks of contiguously methylated sites. For instance, the range of individual methylation efficiencies observed in the presence of DNMT3A on the pBR bottom strand was 28-fold (Figure S4). In sharp contrast, the corresponding range of methylation efficiencies at these sites was 4.3-fold in the presence of DNMT3L, which represents a significant reduction (Figures S4 and S5). Similarly, when this analysis was performed for DNMT3B at the same region, the maximal difference between individual sites shifts from 34-fold in the absence of DNMT3L to 12.5-fold in its presence, a statistically significant reduction (Figure S5). This phenomenon is explained by the fact that the sites that show the strongest stimulation by DNMT3L correspond to those that were the least methylated by DNMT3A or DNMT3B on their own. This is clearly illustrated in Figure 8B by an inverse relationship between the fold stimulation afforded by DNMT3L at each individual CpG site and individual methylation efficiencies in the presence of DNMT3A or DNMT3B alone. The sites with the lowest initial methylation showed a striking 32–35-fold increase by DNMT3L while the sites with the greatest initial methylation only showed a 2–3-fold increase. Altogether this shows that while DNMT3L does not alter the intrinsic sequence preference of DNMT3A and DNMT3B, its stimulatory effect is most felt at the sites with the initial lowest methylation efficiency. DNMT3L therefore attenuates the intrinsic sequence preference of DNMT3A and DNMT3B, resulting in the deposition of more uniform methylation patterns.

## Discussion

One important unanswered issue surrounding the establishment of DNA methylation patterns relates to the individual contribution of each member of the *de novo* DNA methyltransferase family to the actual formation of the patterns *in vivo.* Here, we took advantage of an episomal assay and examined *de novo* methylation mediated by DNMT3A or DNMT3B (with and without DNMT3L) in human cells using extensive bisulfite methylation sequencing. This assay offers several advantages. The cell line that we used, which has little or no endogenous *de novo* methylation activity [44], allowed us to study the activity of exogenously expressed full-length human DNMT3A and DNMT3B separately, an otherwise impossible task when analyzing genomic DNA methylation profiles. Likewise, using unmethylated episomes as substrates for DNA methylation allowed us to ensure that the patterns deposited by each enzyme were relatively unaffected by pre-existing parameters such as chromatin compaction, composition, and modification states. Such parameters are unavoidable when studying genomic methylation patterns and strongly compound the activity of *de novo* DNMTs. While representing a simpler substrate than genomic DNA, the episomes used here are biologically relevant in that they are self-replicating, acquire chromatin, and undergo maintenance DNA methylation and epigenetic silencing [30], [45]. In addition, episomal *de novo* methylation requires the SNF2-family chromatin remodeling factor Lsh and responds to the DNMT3L stimulatory factor just as observed at endogenous loci [40], [46]. Therefore, this assay offers a useful window into the intrinsic *de novo* activity of each enzyme *in vivo*.

Our data indicate that the human DNMT3A and DNMT3B enzymes instruct the deposition of unique DNA methylation patterns. These patterns are characterized by clear and reproducible high and low methylation sites distinguished by greater than 10-fold differences in individual methylation efficiencies. This indicates that DNMT3A and DNMT3B do not methylate DNA at random. For DNMT3A, the overall difference in methylation efficiency between the top 10% most methylated sites and the bottom 10% least methylated sites was 5.3-fold (Figures 1 and 2). This difference was 8.3-fold for DNMT3B, which consistently appeared more selective than DNMT3A in its DNA methylation patterns (Figure 3 and 4). The selectivity of *de novo* methylation measured here for human DNMT3A and DNMT3B is in agreement with data from *A. thaliana* for which hotspots and coldspots varied up to 13-fold in methylation efficiency depending on sequence context [26], [27]. Sequence alignments revealed that specific residues were significantly over-represented at particular positions flanking “hot” or “cold” CpG sites. Importantly, the motifs revealed by such alignments were (i) reproducible across independent biological replicates; (ii) reproducible across active isoforms of DNMT3A and DNMT3B; (iii) derived from the alignment of multiple, carefully selected, hotspots and coldspots picked from over 270 distinct CpG sites originating from several test regions of various provenance; (iv) recapitulated upon analysis of DNA methylation patterns *in vitro* for DNMT3A; and (v) validated by an independent enzyme-based assay for DNMT3B. Altogether, this indicates that these motifs represent high confidence assignments.

The motifs associated with these hotspots and coldspots showed a clear and consistent “mirror image” pattern of enrichment at specific positions. For instance, in the case of DNMT3A, highly methylated CpG sites showed a significant over-representation of a T at position −2, while poorly methylated sites showed enrichment for an A at this position. To a lesser degree, hotspots tended to show over-representation for a C at position +2 while coldspots tended to carry a G at this position (Figure 2). A similar observation was made for DNMT3B: hotspots were significantly enriched for a G at position +1, while coldspots displayed a C (Figure 4). The observation of such patterns of reciprocal enrichment between hotspots and coldspots strongly suggests that the positions identified in our study are key in the selection of good or bad flanks. In the case of DNMT3B, the observation that HhaI sites (GCGC) represent cold sites compared to HpaII sites (CCGG) (Figure 4), additionally suggests that most of the variation in DNA methylation efficiency was captured by the −1 and +1 positions since these restriction enzymes are not sensitive to variation outside their recognition site. Altogether, our data indicate that DNMT3A and DNMT3B discriminate between good and bad flanks by responding to the sequence composition at distinct positions around the CpG site. While DNMT3A responds to the composition at the −2 and + 2 positions, DNMT3B mediates its selection through the −1 and +1 positions. This likely reflects intrinsic differences in the catalytic properties of DNMT3A and DNMT3B and suggests that DNMT3A and DNMT3B, despite their strong amino acid conservation over the catalytic domain, contact DNA around the target CpG site differently.

Interestingly, the −2, −1, +1 and +2 positions flanking the CpG site were the only positions to show statistically significant deviations in their sequence composition. These observations are in agreement with data from *A. thaliana* for which selection for a good or bad flank was essentially mediated through the two positions adjacent to the target site [26], [27]. Our analysis did not uncover any significant relationship between CpG spacing and DNA methylation efficiency and, sites separated by 8–10 bp did not appear more efficiently methylated. In contrast, we observed several instances of strong methylation hotspots that were not flanked by any CpG site within 8–10 bp. Likewise, we observed in a few instances that the presence of a strong methylation hotspot did not necessarily translate into similarly high methylation efficiency for a neighboring CpG site located 8–10 bp further (data not shown). This suggests that, at least in the context of our experimental system, the proposed relationship between CpG spacing and DNA methylation efficiency [47] may not apply or may be compounded by other effects. When compared to prior studies examining possible site-preference by using purified DNMT3A or DNMT3B proteins in *in vitro* methylation assays, our results are in close agreement with data from Lin *et al.*, (2002) [29]. In this study, the authors reported that the full length murine DNMT3A protein shows elevated activity at sites carrying pyrimidines at positions −2 and +1. Such preference is in remarkable agreement with our *in vitro* data using human DNMT3A (Figure 2 and Figure S3) and is consistent with our *in vivo* evidence. Our data are more difficult to reconcile with the data of Handa *et al.* (2002) who used murine enzymes either full-length or in a truncated C-terminal form and determined that both DNMT3A and DNMT3B share a common preference for AT-rich flanks and for certain palindromic sequences [28]. In our study, flanks such as 5′-ACGT-3′ or other combinations of A/T bases at the −1 and +1 positions, were consistently found around or slightly below the median in terms of DNA methylation efficiencies. This could represent a difference between murine and human enzymes or between the experimental systems used.

Overall, our study demonstrates that the DNMT3A and DNMT3B *de novo* DNMTs possess clear and distinct flanking sequence preferences *in vivo*. Such preferences, while clearly significant, remain sufficiently relaxed that they are compatible with the methylation of a wide variety of CpG sites, as observed in the genome. It should also be noted that only a portion of all possible flanks have been examined here and that the ultimate identities of the DNMT3A and DNMT3B signature motifs might evolve upon surveying a more complete sequence space. However, several lines of evidence suggest that the signature motifs described here may have predictive value. For instance, we showed that promoter-associated CpG islands showed a depletion for CpG sites corresponding to predicted DNMT3B hotspots and an enrichment for predicted coldspots. In contrast, a distinct set of CpG islands which tend to associate with gene bodies and tend to be methylated did not show evidence for such selection (Figure 6). This suggests that the intrinsic sequence preference of DNMT3B may have contributed to shaping the composition of CpG island promoters to favor the maintenance of a state devoid of DNA methylation. Likewise, we note that recent analysis of the complete human methylome revealed that a T at the −2 position was enriched in high methylation sites in the CHG and CHH context, a type of non-CG methylation that is only observed in pluripotent embryonic stem cells that are characterized by high *de novo* methyltransferase activity [48]. This enrichment is consistent with our motif assignments and with prior studies which implicated DNMT3A in non-CG methylation activity [37], [38].

In this study, we also investigated the effect of the DNMT3L protein on the deposition of DNA methylation patterns. Two main novel findings emerged. First, DNMT3L appears to direct *de novo* methylation towards well-chromatinized DNA templates. This was observed initially as a bias in the DNMT3L stimulatory effect in favor of newly replicated DNA strands (Figure 8). A strand-tagging experiment, however, allowed us to demonstrate that this bias was not due to a direct coupling between DNMT3L-mediated DNA methylation and DNA replication. This conclusion is expected from the fact that DNMT3L mediates the deposition of DNA methylation patterns in non-replicating cell types during germ cell development [42]. We therefore suggest that DNMT3L may require properly chromatinized DNA substrates for its function. This proposal is consistent with the fact that DNMT3L binds to histones directly [18], [19]. In that context, the “replication” bias we observed for DNMT3L likely reflected the necessity for replication-coupled nucleosome deposition to occur on newly transfected episomes. Interestingly, DNMT3A and DNMT3B on their own did not show any “replication” bias even though evidence clearly suggests that these proteins bind to nucleosomes [49], [50]. This suggests that DNMT3L imposes an even stricter requirement for well-chromatinized substrates onto the process of *de novo* DNA methylation. Our second finding showed that while DNMT3L does not appear to affect the intrinsic sequence preference of DNMT3A and DNMT3B, its stimulatory effect is not felt uniformly across CpG sites. On the contrary, our analysis revealed a striking inverse relationship between the stimulation afforded by DNMT3L and the initial DNA methylation efficiency by DNMT3A or DNMT3B (Figure 8). Such an effect could again result from the ability of DNMT3L complexes to associate with chromatin, thus favoring the occupancy of DNA by DNMT3A and DNMT3B. An increased DNA dwell time would greatly increase the likelihood that a methylation coldspot will become methylated without strongly affecting the outcome at a rapidly methylated hotspot. This proposal is consistent with the observation that expression of DNMT3L appears to attenuate the impact of intrinsic flanking sequence preferences of DNMT3A and DNMT3B, lowering the range of individual methylation efficiencies between CpG sites (Figure S5) and triggering the deposition of more uniform patterns characterized by longer methylation tracts (compare Figures S3 and S4). This is in agreement with the *in vivo* function of DNMT3L, which ensures that its multiple target regions (interspersed repeats, satellite repeats, differentially methylated imprinted regions and other chromosomal regions; [15]–[17], [51], [52]) are fully methylated over long blocks of DNA sequence. In that context, it is interesting to note that the drastic reduction of DNA methylation observed in the absence of DNMT3L at imprinting centers may reflect, at least in part, the possibility that such sequences are strongly enriched in methylation coldspots. As discussed above for the *SNRPN* region studied here, imprinting centers overlapping CpG islands tend to exhibit strong GC skew (P.A.G. and F.C., unpublished data). Our data suggests that the C-rich strand of such regions may be particularly difficult to methylate, thus rendering the action of DNMT3L all the more critical at these regions. Altogether, our study reports that the catalytic activity of DNMT3A and DNMT3B show significant and distinct flanking sequence preference *in vivo* and suggests that the ability of DNMT3L to bind to chromatin, in addition to its ability to stimulate the catalytic activity of DNMT3A and DNMT3B, are key to its biological function.

## Materials and Methods

### Expression vectors, episomes, and target sequences

Full-length human DNMT3A, DNMT3A2, DNMT3B (the DNMT3B1 isoform was used throughout, unless indicated) and DNMT3L proteins were expressed in HEK293c18 cells using previously described vectors [41].

The *E. coli dcm* methyltransferease gene (GenBank accession number: YP_853012) was amplified from *E. coli* genomic DNA (DH10B strain) with a forward primer containing an in-frame EcoRI site (underlined) (DcmFOR: 5′-TTTTTTGAATTCATGCAGGAAAATATATCAGT-3′) and a reverse primer containing a BamHI site (underlined) located immediately after the stop codon (DcmREV: 5′-TTTTTTGGATCCTTATCGTGAACGTCGGCCAT-3′). The amplified *dcm* PCR fragment was then digested with EcoRI and BamHI, cloned into the corresponding sites of pcDNA3/Myc [41] and sequence verified. A nuclear localization signal (NLS) was subsequently cloned into the EcoRI site in frame using two annealed oligonucleotides: 5′-AATTCCCCAAGAAAAAGAGGAAAGTCC-3′ and 5′-GGGGTTCTTTTTCTCCTTTCAGGTTAA-3′. The resulting construct, pcDNA3/Myc-dcm, expresses an N-terminally MYC-tagged version of the *E. coli* Dcm methyltransferase carrying a functional NLS.

The pFC19 target episome was used as a methylation target and has been previously described [40]. pFC19 contains the EBNA1/OriP replication system derived from the Epstein-Barr virus and can be stably maintained in mammalian cells. It carries a 940 bp fragment from the differentially methylated region of the human *SNRPN* CpG island. The first two regions to be analyzed corresponded to sequences present on the episomal backbone, namely: (1) a ∼500 base-pair (bp) region of the pBR322 backbone carrying 48 CpG sites; (2) a ∼500 bp of the Hygromycin (Hygro) resistance gene carrying 47 CpG sites. A ∼300 bp region of the *SNRPN* region carrying 23 CpG sites was also analyzed. An additional ∼500 bp region from the human *TIMELESS* CpG island promoter was also cloned instead of the *SNRPN* sequence and analyzed. All sequences are available in Text S1.

### Cell culture, transfections, and DNA recovery

The HEK293 EBNA1 cell line (293c18, ATCC) was used in all experiments and grown under standard conditions. Transfections were performed using either the calcium phosphate method or Turbofect (Fermentas). For each expression vector or episome, 500 ng of DNA was used per well of a 6-well plate. Cells were allowed to grow for 2–3 days after transfection before being transferred to a 100-mm diameter plate. Upon reaching confluence (6–7 days), cells were harvested for episomal DNA extraction according to the Hirt method [53]. No selection was applied. All experiments were conducted at least in duplicate.

For experiments involving a stably replicating pFC19, the episome was first introduced into HEK293c18 cells and the cells were kept under selective pressure (200µg/ml Hygromycin) for over 20 cell divisions. At this point, the pcDNA3/Myc-dcm expression vector was transfected and expression of Dcm methyltransferase was determined every day post-transfection by Western blot using an Anti-MYC antibody (Sigma). Dcm was clearly expressed as early as 24 hours after transfection and expression remained strong for five days, at which point it dropped rapidly (data not shown). The Dcm methyltransferase was clearly active as judged from the fact that genomic DNA extracted 5 days post-transfection was almost entirely resistant to EcoRII, an enzyme that recognizes CC(A/T)GG sites and is sensitive to dcm methylation. DNA from untransfected cells, by contrast, was extensively cleaved (data not shown). Likewise, episomal DNA harvested seven days post Dcm transfection clearly carried dcm methylation as seen by bisulfite methylation sequencing (data not shown). Seven days after transfection with pcDNA/Myc-dcm, DNMT expression vectors in appropriate combinations were introduced and the cells were allowed to grow for another 4–5 days until confluent, at which point episomal DNA was harvested.

### *In vitro* DNA methylation reactions

*In vitro* methylation by the HhaI methyltransferase (New England Biolabs) was performed as recommended by the supplier and verified by restriction enzyme digestion. *In vitro* methylation by DNMT3A was performed using purified recombinant Maltose-Binding Protein (MBP)-tagged DNMT3A catalytic domain (residues 590–912 of human DNMT3A). Reactions were performed using 1µM MBP-DNMT3A and 250 ng of pFC19 DNA in the presence of 100 µM S-adenosyl-L-methionine. The reactions were incubated for 2 hours at 37°C, at which point the proteins were removed by Proteinase K treatment followed by phenol-chloroform extraction and ethanol precipitation.

### Analysis of DNA methylation

Except when indicated, bisulfite methylation sequencing [32] was used systematically to determine the methylation patterns deposited by human *de novo* DNA methyltransferases. For this, the Hirt DNA was first digested with PstI (New England Biolabs) to linearize the DNA or, when desired, by EcoRII (Roche) to enrich for molecules with dcm methylation. Cleavage was followed by sodium bisulfite treatment as described [32] or using the EZ DNA methylation-direct kit (Zymo research). Both strands of DNA were subsequently PCR amplified from different regions of the episome (primers available upon request) and the resulting PCR fragments were cloned using the TopoTA cloning kit (Invitrogen). Single colonies carrying individual DNA molecules were then picked and plasmid DNA sequenced. The overall efficiency of bisulfite conversion in this study was 99.1%. DNA methylation was also analyzed using methylation sensitive restriction enzymes and Southern blot analysis, as described [40]. In the case of the *TIMELESS* and *RNF168* sequences, bisulfite treatment was combined to pyrosequencing in order to extract quantitative and unbiased methylation patterns [54]. Pyrosequencing analysis was conducted by EpigenDx (Worcester, MA).

### Sequence analysis and statistical treatment

To handle and analyze the large amount of bisulfite sequencing information generated in this study, we implemented in-house software programmed in Visual Basic running under a Microsoft Excel environment. The software input consists of a typical ClustalW-type multiple alignment of trimmed sequence data (i.e.. the sequence corresponding to the region under analysis stripped of flanking vector sequence). From this, the software automatically computes the conversion efficiency for each molecule and filters any molecule below a user-defined threshold (no less than 95% conversion in all cases). It then calculates the distribution of methylated and unmethylated CpG sites and reports the data as a standard graph, as shown in Figure 2. The software also calculates the overall methylation efficiency for each DNA molecule and for each CpG site across the analyzed sample. This allows us to rank the various CpG sites according to their individual methylation efficiencies and to extract and align the sequences flanking each CpG, focusing on the top 10% most methylated sites and bottom 10% least methylated sites or any other user-defined portion of the distribution. A statistical test for the enrichment of a residue at any given position above what is expected from the average composition of the sequence being considered is also built-in using a Chi-square test. The statistical significance of enrichment is reflected by a P-value which is calculated from the distribution of Chi-square values. This software is available upon request. Enrichment plots were generated using the WebLogo application package [55].

### Analysis of the distribution of CpG motifs in the human genome

The sequence sets used here correspond to all “specific” CpG islands on human chromosome 1 as defined by Bock and colleagues [39] and accessed from the hg18 build of the UCSC Human Genome Browser (representing a total of 1,033 islands and approximately 1 megabase of DNA sequence). The set of “weak” CpG islands was obtained from the same source but corresponded to CpG islands that have no overlap with specific or balanced CpG islands as defined by Bock and colleagues (a total of 906 islands representing approximately 0.45 megabase of DNA sequence). To evaluate the exceptionality of motif frequencies, we used the R'MES software (http://genome.jouy.inra.fr/ssb/rmes/).This software uses Markovian models to compute the expected distribution of given sequence motifs in a sequence and compares it to actual observations. The score reflects the over- or under-representation of motifs under the model being used.

## Supporting Information

Figure S1 Methylation patterns are similar between full length DNMT3A and DNMT3B and their isoforms, DNMT3A2 and DNMT3B2. For this analysis, the overall methylation pattern at the pBR322 region was determined for each of the 48 CpG sites by combining observations on both the top and bottom strands. The total number of independent samples analyzed was 236 for DNMT3A, 109 for DNMT3A2, 228 for DNMT3B, and 136 for DNMT3B2. A. CpG sites were ranked according to their individual methylation efficiencies. The ranks observed at each of the 48 CpG sites were then plotted against each other for one isoform versus the other, as indicated. A clear correlation was observed, showing that the overall hierarchy of sites is not changed (r^2^ values were 0.71 for DNMT3A and DNMT3A2 (left) and 0.78 for DNMT3B and DNMT3B2 (right)) B. The total number of methylation events normalized to the average of each sample are plotted at each CpG site along the pBR322 region for each isoform. The patterns observed were clearly similar in that high sites remained high and low sites remained low in each case.
(1.73 MB EPS)

Figure S2 High and low methylation efficiency sites at the SNRPN test region (C-strand) conform to the sequence motifs defined for DNMT3A and DNMT3B at the pBR and Hygro test regions. High and low sites for DNMT3A show clear evidence for selection at the −2 and +2 positions (highlighted in yellow) according to the predictive motifs defined for DNMT3A (top panel). Likewise, high and low sites for DNMT3B show clear evidence for selection at the +1 position (highlighted in yellow) according to the predictive motifs defined for DNMT3B.
(1.37 MB EPS)

Figure S3 A. This panel represents the DNA methylation patterns deposited by purified full-length DNMT3A2 enzyme in complex with DNMT3L, as measured by bisulfite methylation sequencing. Symbols are as described above. B. The *in vitro* flanking sequence preference for DNMT3A was determined by focusing on the 10% most and least methylated sites observed in panel A. Both top and bottom sites show evidence of selection for particular residues at the −2 position, as was observed *in vivo*.
(1.60 MB EPS)

Figure S4 *In vivo* methylation patterns observed on the bottom strand of the pBR322 region upon expression of DNMT3A (left) or DNMT3A and DNMT3L (right). The arrows highlight the two most distinct sites in terms of their methylation efficiencies for DNMT3A.
(1.97 MB EPS)

Figure S5 DNMT3L promotes the formation of more uniform DNA methylation patterns. The y-axis describes the overall range of methylation efficiencies observed among 48 different CpG sites on the pBR322 bottom strand. In all cases, the least methylated site within a sample is given an arbitrary value of 1. The whisker plot (box area comprises 75% of all values) shows that DNMT3L significantly reduces the spread in methylation efficiencies (***, pvalue<0.001 according to unpaired t-test).
(1.28 MB EPS)

Text S1 Sequence files for analyzed regions.
(0.03 MB DOC)
