# Supplementary material for: DNMT3L Modulates Significant and Distinct Flanking Sequence Preference for DNA Methylation by DNMT3A and DNMT3B In Vivo
Source: PLoS Genet. 2010 Sep 9;6(9):e1001106. doi: 10.1371/journal.pgen.1001106 (PMC2936528; doi:10.1371/journal.pgen.1001106)
Supplement: Text S1 — Sequence files for analyzed regions. (0.03 MB DOC) [file pgen.1001106.s006.doc]

*Supplementary Information*: sequences of regions analyzed by methylation bisulfite sequencing in this study. CpG sites are indicated in bold underlined fonts. Only one strand is shown.

SNRPN REGION

ggtcacaggaaaattaaaggctg**CG**gactgtgctactgccccttctgatgccccctcctctacacagcaatcattcag**CG**tcccttagtcactc**CG**gacag**CG**acaggccc**CGCG**gc**CG**ccatgcccac**CG**cctccatgccatgcccac**CG**c**CG**ccatgcctac**CG**c**CG**ccaaagtccaccac**CG**ccatgcctacc**CG**ctgccaatgcccac**CG**c**CG**ccaatacccactgt**CG**c**CG**ccttccccctacctcccagccacttccta**CG**gactctccc**CGCG**c**CGCG**accaccaacacaacccccaccactgtcacac**CG**actcatccccctggtccactgccatagcctcct**CG**cct**CG**gtcactg**CG**a**CG**aattccccccccagt**CG**cccca**CG**taccctgctccacca**CG**cagtggtcactattatacacctacctg**CG**ctcaacaccccctaaatac**CG**atcacttca**CG**tacctt**CG**ccc**CG**ccacaatcactccaatatacctacctc**CG**cctaaaatccctatgcactggt

pBR REGION

tgcacacagcccagcttggag**CG**aa**CG**acctacac**CG**aactgagatacctacag**CG**tgagctatgagaaag**CG**cca**CG**cttcc**CG**aagggagaaagg**CG**gacaggtatc**CG**gtaag**CG**gcagggt**CG**gaacaggagag**CG**ca**CG**agggagcttccagggggaaa**CG**cctggtatctttatagtcctgt**CG**ggttt**CG**ccacctctgacttgag**CG**t**CG**atttttgtgatgct**CG**tcagggggg**CG**gagcctatggaaaaa**CG**ccagcaa**CGCG**gccttttta**CG**gttcctggccttttgctggccttgaagctgtccctgatggt**CG**tcatctacctgcctggacagcatggcctgcaa**CGCG**ggcatcc**CG**atgc**CG**c**CG**gaag**CG**agaagaatcataatggggaaggccatccagcct**CGCG**t**CGCG**aa**CG**ccagcaaga**CG**tagcccag**CGCG**t**CG**gccc**CG**agatg**CG**c**CGCG**tg**CG**gctgctggagatgg**CG**ga**CGCG**atggatatgttctgccaagggttggtttg

HYGRO REGION

cataaaccattccccatgggggaccc**CG**tccctaaccca**CG**gggccagtggctatggcagggcttgc**CG**ccc**CG**a**CG**ttggctg**CG**agccctgggccttcacc**CG**aacttgggggttggggtggggaaaaggaagaaa**CGCG**gg**CG**tattggccccaatggggtct**CG**gtggggtat**CG**acagagtgccagccctgggac**CG**aaccc**CGCG**tttatgaacaaa**CG**acccaacacc**CG**tg**CG**ttttattctgtctttttattgc**CG**tcatag**CGCG**ggttccttc**CG**gtattgtctccttc**CG**tgtttcagttagcctcccccatctcc**CG**atcccca**CG**agtgctgggg**CG**t**CG**gtttccactat**CG**g**CG**agtacttctacacagccat**CG**gtccaga**CG**gc**CGCG**cttctg**CG**gg**CG**atttgtgta**CG**cc**CG**acagtcc**CG**gctc**CG**gat**CG**ga**CG**attg**CG**t**CG**cat**CG**accctg**CG**cccaagctgcatcat**CG**aaattgc**CG**tcaaccaagctctgatagagttggtca

TIMELESS 500 (the region analyzed by pyrosequencing is highlighted).

AAAAGGGACTCAG**CG**TTTCC**CG**agaatgcccccatagcTT**CG**aaaggatccc**CG**tgTC**CG**CttAG**CG**Ccctct**CG**Ccacacactcactcacc**CG**Ctccctg**CGCG**tcctcagaagcc**CG**CaggagccAC**CG**Cccctctgg**CGCGCG**Gc**CG**cagccttTC**CG**CCaccaggctcagctggAC**CG**Ctccc**CG**CcTG**CGCG**aagAG**CG**agg**CGCG**G**CG**agc**CGCG**ctggtcca**CG**tgacTC**CG**AG**CG**aactgggg**CG**CagattggtgaagaaggaaggaaaagggaAG**CG**agtgcagggactgttttatgtgaggt**CG**atctaaaaatcacAG**CG**CtatgaattttctgctgaattatagtagaatcaaagTC**CG**attagaggtgaatttacaaacattcttccccttcttctagtcaAG**CG**CactattccaagtcttaaatagttagttagaaaagttaggttttatttgggaggaagtaaaGAGCTGAAGTACAGGGAGGAA
